# Body Weight Perception and Other Factors Associated with Overweight and Obesity in U.S. Adolescents

**DOI:** 10.3390/children12020169

**Published:** 2025-01-29

**Authors:** Gulzar Shah, Indira Karibayeva, Padmini Shankar, Semon Mason, J. Michael Griffin

**Affiliations:** 1 Department of Health Policy and Community Health, Jiann-Ping Hsu College of Public Health, Georgia Southern University, Statesboro, GA 30460, USA; gshah@georgiasouthern.edu (G.S.); jg36146@georgiasouthern.edu (J.M.G.); 2 School of Health & Kinesiology, Georgia Southern University, Statesboro, GA 30460, USA; pshankar@georgiasouthern.edu; 3 Department of Nutrition, University of Bridgeport, Bridgeport, CT 06604, USA; semason@my.bridgeport.edu

**Keywords:** adolescents, BMI, overweight, obesity, Youth Risk Behavior Surveillance System (YRBSS), perceived weight status

## Abstract

Background/Objectives: This study examines the factors associated with U.S. adolescents’ obesity and overweight status. Methods: Using a multivariable logistic regression, we analyzed the data from the 2021 Youth Risk Behavior Surveillance System (YRBSS), comprising 17,232 students. Results: The odds of being obese or overweight were significantly higher (*p* ≤ 0.05) for the participants who perceived themselves as slightly overweight (AOR, 13.31; 95% CI [11.83, 14.97]) or very overweight (AOR, 39.29; 95% CI [30.12, 51.25]) compared to those who perceived their weight as about right. The participants with significantly higher odds included those aged 14 years (AOR, 2.53) compared to those aged 13 years or younger; male students (AOR, 1.63) compared to female students; and American Indian/Alaska Native and Native Hawaiian/Other Pacific Islander students (AOR, 2.11), Black or African American students (AOR, 2.63), Hispanic/Latino students (AOR, 1.54), and students of multiple races (AOR, 1.56), compared to White students. The odds were also significantly higher for the participants who did not eat breakfast on all seven days of a week (AOR, 1.21) and for the students who did not report their mental health status (AOR, 2.07) compared to those who reported their mental health as mostly or always not good. Conclusions: These findings suggest schools are uniquely positioned to implement strategies for healthier behaviors designed and implemented with a focus on health equity.

## 1. Introduction

Adolescent obesity in the United States has escalated to critical levels, presenting substantial public health challenges. Recent statistics reveal that approximately 20% of U.S. adolescents are classified as obese, with a higher prevalence among Hispanic (26.2%) and non-Hispanic Black (24.8%) youth compared to their non-Hispanic White counterparts (16.6%) [1]. Since the inception of the Youth Risk Behavior Surveillance System (YRBSS) in 1999, there has been a consistent escalation in the number of overweight and obese adolescents, with 16% of adolescents being at risk of becoming overweight in 1999 [2]. This upward trend is alarming due to its association with numerous adverse health outcomes, including type 2 diabetes, cardiovascular diseases, and mental health conditions [3].

An individual’s self-assessment of their body weight, known as their body weight perception, significantly influences an adolescent’s health behaviors and weight management strategies [4,5]. Body weight perception influences one’s actions and activities targeting weight management, because an underestimation of weight may lead to a lack of action, as evidenced by studies showing that adolescents who misperceive their weight status are less likely to engage in appropriate weight-related health behaviors [6,7,8]. For instance, those who underestimate their weight may not recognize the need for weight management, while those who overestimate may engage in unnecessary or harmful weight control practices [9,10,11]. Furthermore, weight misperception has been associated with psychological distress, such as decreased self-esteem and increased depressive symptoms [12,13].

Adolescence is a crucial phase for developing these perceptions, as peer influence, cultural standards, and the prevalence of social media amplify the pressures regarding one’s appearance [14]. Demographic factors such as age, sex, and race/ethnicity significantly influence both the actual weight status and perceived weight among adolescents [15,16]. Research suggests that older adolescents and females are prone to perceive themselves as overweight, regardless of their actual weight [11,17]. Moreover, cultural differences across racial and ethnic groups can affect body image ideals and perceptions, potentially influencing weight-related behaviors and outcomes. Notably, obesity rates are more prevalent among Black or African American (21.2%) and Hispanic (20.2%) adolescents compared to White (13.7%) and Asian (7.7%) adolescents, as reported in the 2021 YRBSS report [18].

Lifestyle behaviors, including dietary habits and physical activity levels, are major determinants of adolescents’ weight status [19]. An inadequate consumption of fruits and vegetables, an excessive consumption of sugar-sweetened beverages, and insufficient physical activity have been consistently linked to an increased risk of overweight and obesity in this population [20,21]. While there has been a decrease in soda consumption, there has not been a significant increase in the consumption of plain water, as adolescents are opting for other flavored beverages, such as fruit and sports drinks [22]. Moreover, sedentary behaviors, such as excessive screen time, further exacerbate the risk. Studies show a continuous decline in physical activity and an increase in screen time; the Adolescent Brain Cognitive Development study indicates that the mean daily screen time among adolescents was six and a half hours [23]. The influence of electronics and screen time has significantly contributed to the sedentary behavior of young children and adolescents [24].

Utilizing data from the 2021 Youth Risk Behavior Survey (YRBS), this research investigated the factors associated with overweight and obesity among U.S. adolescents [18]. Specifically, this study explored the association of perceived weight, demographic variables, and health-related behaviors with overweight and obesity among adolescents. Our primary hypothesis was that adolescents’ perception of their weight being an issue may have led to weight management behaviors aimed at addressing overweight and obesity. These insights are critical for developing targeted public health initiatives aimed at mitigating the obesity epidemic among adolescents in the United States.

## 2. Materials and Methods

### 2.1. Study Design and Data Source

This study employs cross-sectional survey data from the YRBSS [18]. The 2021 YRBS, a nationally representative survey, was conducted by the Centers for Disease Control and Prevention (CDC). The 2021 YRBS utilized a three-stage cluster sampling methodology to ensure a nationally representative sample of high school students (grades 9 through 12). The sampling frame included all standard public schools, Catholic schools, and other private institutions with at least 40 enrolled students across the 50 U.S. states and the District of Columbia. It was developed using data obtained from Market Data Retrieval and the National Center for Education Statistics. In the first stage, geographic primary sampling units were selected using probability proportional to their population size. During the second stage, schools were selected from the primary sampling units, with a probability proportional to their enrollment size. Invitations were sent to relevant school boards or district authorities to secure permission for school participation before selection. In the third stage, classes within the selected schools were randomly selected [25]. Of the 209 schools sampled, 152 participated, yielding a school response rate of 72.7%. Classes were selected using systematic equal probability sampling with a random start. Students from the chosen classes were all invited to take the survey, resulting in 17,232 final valid responses out of 21,791 students participating, corresponding to a student response rate of 79.1%. Consent protocols for the 2021 YRBS were administered in accordance with CDC guidelines and the specific requirements of local and state authorities. The final response rate was 57.5%. The survey used self-administered, anonymous questionnaires completed during regular class periods.

The inclusion criteria for this analysis consisted of students enrolled in grades 9–12 in public, Catholic, and private schools across the U.S. who completed the 2021 YRBS survey during the study period. Students with biologically implausible values were excluded, following CDC recommendations for ensuring data quality.

### 2.2. Measures

Supplemental Table S1 (Table S1) provides a detailed summary of the variables analyzed in this study, including response scales, collapsed categories, and corresponding YRBS identifiers, according to the YRBS data users guide [25]. The primary dependent variable was a dichotomous measure categorized as underweight/normal versus overweight/obese. This classification is based on Body Mass Index (BMI) and BMI percentiles. BMI was calculated using participants’ self-reported weight and height with the formula: BMI = (weight in pounds × 703)/(height in inches)^2^. BMI percentiles, adjusted for age and sex, were determined using the SAS Program for CDC Growth Charts, which was developed by the Division of Nutrition, Physical Activity, and Obesity at the CDC, and were included in the dataset [26].

The independent variables were categorized into three groups: perceived weight status, demographic variables, and behavioral and other factors. Participants’ self-perceived weight status was assessed with the question: “How do you describe your weight?” Responses were categorized into five groups: very underweight, slightly underweight, normal weight, overweight, and obese.

Demographic variables included age, gender, and race/ethnicity. Age was assessed by the question, “How old are you?” with response options categorized into six groups: 13 years old or younger, 14 years old, 15 years old, 16 years old, 17 years old, and 18 years old or older. Gender was assessed by the question, “What is your sex?” with two response options: female and male. Race was evaluated by the question, “What is your race?” with five response options: American Indian or Alaska Native, Asian, Black or African American, Native Hawaiian or Other Pacific Islander, and White. Ethnicity was assessed by the question, “Are you Hispanic or Latino?” with response options of yes or no. For the analysis, race and ethnicity were combined into the following categories: White, Asian, Black or African American, Hispanic/Latino, American Indian/Alaska Native/Native Hawaiian/Other Pacific Islander, multiple race—Hispanic/Non-Hispanic, and did not report.

Behavioral independent variables included dietary habits and physical activity. Dietary habits were evaluated by several questions: fruit consumption was assessed by “During the past 7 days, how many times did you eat fruit?”; fruit juice consumption by “During the past 7 days, how many times did you drink 100% fruit juices such as orange juice, apple juice, or grape juice? (Do not count punch, Kool-Aid, sports drinks, or other fruit-flavored drinks.)”; vegetable consumption by “During the past 7 days, how many times did you eat other vegetables? (Do not count green salad, potatoes, or carrots.)”; soda or pop consumption by “During the past 7 days, how many times did you drink a can, bottle, or glass of soda or pop, such as Coke, Pepsi, or Sprite? (Do not count diet soda or diet pop.)”; and milk consumption by “During the past 7 days, how many glasses of milk did you drink? (Count the milk you drank in a glass or cup, from a carton, or with cereal. Count the half-pint of milk served at school as equal to one glass.)”. Response options for these questions included categories such as did not eat/drink, 1 to 3 times, 4 to 6 times, 1 time per day, 2 times per day, 3 times per day, and 4 or more times per day. Eating breakfast was assessed by the question, “During the past 7 days, how many days did you eat breakfast?” with response options ranging from 0 days to 7 days. Physical activity was assessed by the question, “During the past 7 days, on how many days were you physically active for a total of at least 60 min per day? (Add up all the time you spent in any kind of physical activity that increased your heart rate and made you breathe hard some of the time.)” with response options from 0 days to 7 days. For the analysis, the response options for behavioral questions were recoded into the following categories: yes, no, and did not report.

Other independent variables included mental health, sleep duration, and sleeping in their parent’s or guardian’s home. Mental health was assessed by the question, “During the past 30 days, how often was your mental health not good? (Poor mental health includes stress, anxiety, and depression.)” with response options ranging from never to always. For the analysis, this was recoded as “Reported that their mental health was most of the time or always not good,” with three response options: yes, no, and did not report. Sleep duration was assessed by the question, “On an average school night, how many hours of sleep do you get?” with seven response options: 4 h or less, 5 h, 6 h, 7 h, 8 h, 9 h, and 10 or more hours. For the analysis, this was simplified to “Got 8 or more hours of sleep (on an average school night)” with three response options: yes, no, and did not report.

Finally, sleeping arrangements were assessed through the question, “During the past 30 days, where did you usually sleep?” with response options: in my parent’s or guardian’s home; in the home of a friend, family member, or other person because I had to leave my home or my parent or guardian cannot afford housing; in a shelter or emergency housing; in a motel or hotel; in a car, park, campground, or other public place; I do not have a usual place to sleep; and somewhere else. For the analysis, this was recoded as “Did not sleep in their parent’s or guardian’s home (during the 30 days before the survey)” with three response options: yes, no, and did not report.

### 2.3. Statistical Approach

Descriptive statistics were employed to summarize the frequency distributions of all study variables. To investigate the relationship between perceived weight status and actual weight status based on BMI, and to identify demographic and behavioral predictors of the dichotomous variables of being overweight or obese, we conducted multivariable logistic regression analyses. For each predictor, adjusted odds ratios (AORs) and 95% confidence intervals (CIs) were calculated, with statistical significance set at *p* < 0.05. The AORs indicate that the odds for a certain attribute of an independent variable in comparison with its reference category reflect comparative odds after controlling for all other explanatory variables included in the model. All statistical analyses were performed using IBM SPSS Statistics, version 20.0 [27]. Georgia Southern University Institutional Review Board approved this study, protocol #H25043, dated 28 August 2024.

## 3. Results

### 3.1. Descriptive Statistics

Table 1 presents the frequency distributions of the variables of interest. The information on the height and weight of 14,896 students was available for BMI calculations, and 4915 (32.4%) were classified as overweight or obese. Out of the 12,275 students who provided answers regarding their self-described weight, 3981 (32.3%) described themselves as slightly or very overweight. The greatest proportion of the study sample was 15 years old (25.3%), male (51.7%), and White (49.5%). The distribution of behavioral factors was as follows: the largest proportion of the students (47.7%) ate fruit or drank 100% fruit juices one or more times per day; 47.2% ate vegetables one or more times per day; 76.9% did not drink a can, bottle, or glass of soda or pop one or more times per day; 60.0% did not drink one or more glasses of milk per day; 69.9% did not eat breakfast on all seven days before the survey; 73.4% were not physically active for at least 60 min per day on all seven days; 63.7% did not report that their mental health was mostly or always not good; 69% did not get eight or more hours of sleep on an average school night; and 88.8% slept in their parent’s or guardian’s home during the 30 days before the survey.

### 3.2. Logistic Regression Analysis Results

Table 2 presents the multivariable logistic regression analysis results, highlighting the significant factors contributing to the students being overweight or obese. The odds of being obese or overweight were significantly lower for the participants who perceived themselves as slightly underweight (AOR = 0.17; 95% CI 0.13, 0.21) or very underweight (AOR = 0.51, 95% CI [0.37, 0.71], *p* < 0.001) compared to those who perceived their weight as about right. Conversely, the odds of being obese or overweight were significantly higher for the participants who perceived themselves as slightly overweight (AOR = 13.31, 95% CI [11.83, 14.97], *p* < 0.001) or very overweight (AOR = 39.29, 95% CI [30.12, 51.25], *p* < 0.001) compared to those who perceived their weight as about right. The odds of being obese or overweight were significantly higher for the participants aged 14 years (AOR = 2.53, 95% CI [1.08, 5.95], *p* = 0.03) compared to those aged 13 years or younger. The odds of being obese or overweight were significantly higher for male students (AOR = 1.63, 95% CI [1.45, 1.82], *p* < 0.001) compared to female students.

The odds of being obese or overweight were significantly higher for American Indian/Alaska Native, Native Hawaiian/Other Pacific Islander students (AOR = 2.11, 95% CI: [1.31, 3.41], *p* = 0.002), Black or African American students (AOR = 2.63, 95% CI [2.27, 3.04], *p* < 0.001), Hispanic/Latino students (AOR = 1.54, 95% CI [1.26, 1.88], *p* < 0.001), and students of multiple races (AOR = 1.56, 95% CI [1.36, 1.80], *p* < 0.001) compared to White students. Conversely, the odds of being obese or overweight were significantly lower for Asian students (AOR = 0.45, 95% CI [0.33, 0.60], *p* < 0.001) compared to White students.

Among the behavioral factors, the odds of being obese or overweight were significantly higher for the participants who did not eat breakfast on all seven days before the survey (AOR = 1.21, 95% CI [1.06, 1.38], *p* = 0.005) compared to those who did eat breakfast. Additionally, the odds of being obese or overweight were significantly higher for the students who did not report their mental health status (AOR = 2.07, 95% CI [1.19, 3.61], *p* = 0.010) compared to those who reported that their mental health was mostly or always not good.

## 4. Discussion

The data derived from the 2021 YRBS were analyzed to highlight the factors associated with overweight and obesity in U.S. high school students, including perceived weight, demographic, behavioral, and other factors. The results indicate that based on BMI, 32.4% of the students were classified as overweight or obese. This is a significant finding, considering these numbers are much higher than the 22.2% national prevalence between 2017 and 2020, as reported by the CDC in 2021 [1]. The health consequences of childhood overweight and obesity escalating to chronic diseases in adulthood are well-reported [28,29]. Further intensifying this public health catastrophe, the discrepancies observed in the misperception of body weight compared to actual high body mass index promote risky lifestyle behaviors and exacerbate poor health outcomes among adolescents.

One’s perceived weight status emerged as a strong predictor. Our study showed that compared to the participants who perceived their body weight to be right, those who perceived themselves as underweight demonstrated a lower likelihood of being overweight or obese, and those who perceived themselves as overweight were more likely to be overweight or obese among this study sample. These findings suggest that the students had realistic perceptions of their weight status relative to their actual weight. This contrasts with another study where one-fifth of the adolescents misperceived their body weight, with the females leaning more towards an overestimation and the males towards an underestimation [30]. Our results also do not corroborate the WHO’s suggestion that adolescents may be out of touch regarding their self-perception of body weight. An analysis of 745,000 adolescents from 41 countries to study, over time, the trends in body weight perception revealed an elevation in the underestimation and a reduction in the overestimation of one’s weight status [31]. Additionally, our results on the students’ weight perception show a promising change from the YRBS 2015 data, which reported that 22.9% of the high school students had some form of weight misperception that was highly correlated with an unhealthy diet and behavior [30]. Multiple study results show that discrepancies in weight perception can have far-reaching implications from a clinical and public health context since a mismatch between one’s body weight perception and one’s actual weight can perpetuate unhealthy lifestyle behaviors among adolescents and negatively impact their mental health [11,31,32,33,34,35,36,37,38,39,40,41].

The demographic variables also played a significant role. The male adolescents exhibited higher odds of being classified as overweight and obese compared to the females, aligning with national trends indicating a higher obesity prevalence among male youths. Racial and ethnic disparities were evident; Black or African American, Hispanic/Latino, American Indian/Alaska Native adolescents, and those who did not report their race or ethnicity had higher odds of being classified as overweight and obese compared to their White counterparts, reflecting persistent health disparities that warrant targeted public health interventions [42].

The health-related behaviors further contributed to weight status. Skipping breakfast was associated with a higher likelihood of the adolescents being overweight or obese, highlighting the importance of regular meal patterns in weight management. Adolescence is a period of rapid growth, when energy and nutrient needs are high. Body image dissatisfaction stemming from a misperception of one’s body weight may influence dietary choices, leading to unhealthy dietary choices and disordered eating habits [43]. Our results show a weighted percent of 69.9% of students did not eat breakfast on all seven days before the survey and they were more likely to be overweight or obese compared to those who ate breakfast, and this was an increase from 66.9% reported in the 2019 YRBS results. Kun Wang et al. reported on the protective effect of breakfast in preventing obesity among adolescents in their meta-analysis [44]. Furthermore, the lack of breakfast promoted a reduced intake of critical nutrients and a more robust intake of fat-rich snacks, increasing the risk for high cholesterol levels [44].

The intricate relationship between mental health and obesity among U.S. adolescents is well-documented. Studies indicate that adolescents with obesity are more susceptible to behavioral disorders, including depression and anxiety. Conversely, those experiencing poor mental health may be at an elevated risk of developing obesity [45]. Our findings reveal that approximately 10% of adolescents did not report on their mental health status, and not reporting on mental health was a significant predictor of overweight and obesity. This lack of reporting could be attributed to various factors, such as stigma, lack of awareness, or the absence of noticeable symptoms. Notably, a study examining Canadian youth found heightened levels of anxiety and depression among those who perceived themselves as overweight, with this trend being particularly pronounced among females [14]. Similarly, the perception of being underweight has been linked to increased anxiety and depression levels, especially among males. This suggests that body weight perception, regardless of one’s actual weight status, plays a significant role in an adolescent’s mental health. The internalization of societal standards and peer comparisons may contribute to these psychological challenges [46].

The COVID-19 pandemic likely influenced the behavioral patterns and health outcomes of adolescents, adding complexity to the interpretation of the 2021 YRBS data. Disruptions in daily life due to school closures, social isolation, and changes in physical activity and dietary habits may have contributed to an increased overweight and obesity prevalence during this period, according to our analysis results. Emerging studies have highlighted declines in physical activity and increases in screen time among adolescents during the pandemic, which are known risk factors for weight gain. In May 2020, the average screen time of U.S. adolescents was estimated to be 7.7 h per day, and only 8.9% of the surveyed adolescents met the guidelines for physical activity levels—a twofold decrease compared to the pre-pandemic level of physical activity—according to the results of the Adolescent Brain Cognitive Development Survey [47,48]. Similarly, the pandemic’s impact on mental health, characterized by heightened levels of stress, anxiety, and depression, could have exacerbated behaviors that promote weight gain, such as emotional eating and decreased physical activity [49].

This study has some limitations that are inherent in studies based on self-reported data in cross-sectional studies conducted amid the COVID-19 pandemic. First, this study used cross-sectional data, which can only support the testing of associations without establishing any causal connections. Second, the self-reported data were used for measures potentially affected by social desirability bias, including height, weight, dietary habits, and physical activity. Third, some self-reported data could have also suffered from recall bias, including fruit and vegetable consumption frequency. Although this study used self-reported data from the 2021 YRBS, which potentially may have been affected by biases, the CDC employed standardized instrument validation tools to minimize these biases in the reported primary variables, including height and weight. Fourth, a limited set of socioeconomic status indicators was available, but poor socioeconomic status conditions could curtail an individual’s ability to afford healthy food and physical activity opportunities. So, it would have been helpful to include those socioeconomic status indicators in our regression analysis to minimize the impact of these confounding factors. Fifth, the COVID-19 must have impacted the data quality of the 2021 YRBS, conducted during this pandemic marked by disruptions in daily life due to social isolation and other restrictions, While the pre-pandemic data indicated a steady rise in adolescent overweight and obesity, comparative analyses with pre-pandemic trends could provide further insights into the pandemic’s specific impact. Comparing pre-pandemic trends in adolescent overweight and obesity with COVID-19 pandemic-related overweight and obesity trends in U.S. adolescents was outside the scope of this study. Additionally, this study relies on secondary data, and the authors of the present manuscript did not have control over the response categories provided to the respondents, such as the perceived weight response options, nor the survey questions that were administered to the respondents. Another limitation of this study is the lack of pre-calculated variables for underweight and normal weight categories in the 2021 YRBS dataset, which necessitated the combination of these groups in the analysis and restricted the focus to overweight and obesity outcomes as explicitly provided by the CDC. Finally, our analyses did not include interaction terms for the independent variables in the regression models because of this study’s focus on examining the influence of weight perceptions on weight status. Regardless of these limitations, this study contributes actionable scientific evidence that is replicable, generalizable, and robust, given its nationally representative sample, sufficiently comprehensive data, focus on equity and disparities, and policy-relevant findings.

Future research should compare pre-pandemic, pandemic, and post-pandemic data to identify evolving behavioral patterns and persistent risk factors. Incorporating validated measures alongside self-reported data and exploring the efficacy of school-based interventions targeting disparities by gender, race, and ethnicity are critical. Advanced statistical methods, such as structural equation modeling, could further elucidate the complex relationships between mental health, weight perception, and obesity, guiding evidence-based and equitable strategies to address adolescent obesity.

## 5. Conclusions

Our results indicate that 32.4% of the students were classified as overweight or obese based on their BMI. The odds of being overweight or obese were significantly higher among the participants who perceived themselves as slightly or very overweight, those aged 14 compared to students aged 13 or younger, and male students compared to female students. Additionally, higher odds were observed among American Indian/Alaska Native, Native Hawaiian/Other Pacific Islander, Black or African American, Hispanic/Latino, and multiracial students compared to White students. The students who did not eat breakfast on all seven days of the week and those who did not report their mental health status also had higher odds of being overweight or obese compared to those who reported their mental health as mostly or always not good.

Notably, the students’ perception of their weight generally aligned with their actual overweight or obesity status. This implies that obesity control interventions may not exclusively rely on educating adolescents about their weight status but also improving opportunity structures for pursuing healthy eating and lifestyles. The obesity prevention programs may focus on devising customized strategies by gender, given that gender was statistically associated with obesity and overweight. Addressing the social determinants of healthy weight for racial and ethnic minorities is critical because Black, Hispanic, American Indian/Alaska Native, and students of multiple races had higher odds of being overweight or obese compared to White students. These findings may also indicate that the cultural competency of physical education teachers and trainers should also be improved. Given that behavioral factors impact obesity and overweight statuses, schools are uniquely positioned to implement strategies to encourage healthier behaviors through improved nutrition programs, behavioral health education, adequate physical activity programs, and mental health support customized by age and gender. Overall, school-based nutrition and health programs should be designed and implemented with a focus on health equity.

## Figures and Tables

**Table 1:** Descriptive characteristics of the study population.

Variables	N (% Weighted)	95% CI (Weighted)
Weight		
Overweight or obese	4915 (32.4)	30.6–34.2
Self-described perceived weight		
Very underweight	459 (3.3)	2.9–3.7
Slightly underweight	1947 (16.0)	15.0–17.0
About the right weight	5888 (48.4)	47.0–49.9
Slightly overweight	3218 (26.2)	25.2–27.2
Very overweight	763 (6.1)	5.5–6.7
Demographic characteristics		
Age		
13 years or younger	101 (0.6)	0.4–0.9
14 years old	3403 (20.0)	18.4–21.6
15 years old	4427 (25.3)	23.9–26.7
16 years old	4276 (24.7)	23.9–25.5
17 years old	3904 (23.6)	22.4–25.0
18 years or older	1023 (5.8)	5.1–6.7
Gender		
Female	8152 (48.3)	46.6–50.0
Race and Ethnicity		
White	9151 (49.5)	45.9–53.1
Asian	850 (4.8)	3.4–6.8
Black or African American	2322 (11.8)	9.4–14.8
Hispanic/Latino	1213 (9.4)	8.0–11.1
American Indian/Alaska Native/Native Hawaiian/Other P.I.	233 (1.2)	0.8–1.7
Multiple race—Hispanic/Non-Hispanic	3031 (21.0)	19.2–22.9
Did not report	432 (2.3)	2.0–2.6
Behavioral and other factors		
Ate fruit or drank 100% fruit juices one or more times per day		
Yes	6641 (47.7)	44.4–51.1
No	6240 (42.4)	39.6–45.3
Did not report	4351 (9.8)	5.4–17.1
Ate vegetables one or more times per day		
Yes	6458 (47.2)	42.8–51.6
No	5748 (39.0)	35.4–42.8
Did not report	5026 (13.8)	8.3–21.9
Drank a can, bottle, or glass of soda or pop one or more times per day		
Yes	2028 (13.3)	11.9–14.8
No	10,671 (76.9)	71.9–81.2
Did not report	4533 (9.8)	5.8–16.3
Drank one or more glasses per day of milk		
Yes	2298 (19.2)	16.7–21.9
No	7252 (60.0)	52.6–66.9
Did not report	7682 (20.8)	13.0–31.8
Ate breakfast on all 7 days (before the survey)		
Yes	3976 (23.3)	21.2–25.6
No	12,100 (69.9)	66.4–73.2
Did not report	1156 (6.8)	3.5–12.6
Physically active at least 60 min per day on all 7 days		
Yes	4145 (23.0)	22.0–24.2
No	12,507 (73.4)	72.2–24.6
Did not report	580 (3.5)	2.9–4.2
Reported that their mental health was most of the time or always not good		
Yes	3729 (26.3)	24.3–28.4
No	9066 (63.7)	59.7–67.5
Did not report	4437 (10.0)	5.8–16.6
Got 8 or more hours of sleep (on an average school night)		
Yes	3219 (20.3)	18.6–22.1
No	10,396 (69.0)	63.0–74.4
Did not report	3617 (10.7)	5.5–19.9
Did not sleep in their parent’s or guardian’s home (during the 30 days before the survey)		
Yes	419 (2.4)	2.0–3.0
No	12,428 (88.8)	83.9–92.3
Did not report	4385 (8.8)	5.3–14.0

**Table 2:** Multivariable logistic regression analysis of factors associated with being obese or overweight.

Variable	AOR	95% CI	*p*-Value
Self-described (perceived) weight (Ref. About the right weight)			
Very underweight	0.51	0.37–0.71	<0.001
Slightly underweight	0.17	0.13–0.21	<0.001
Slightly overweight	13.31	11.83–14.97	<0.001
Very overweight	39.29	30.12–51.25	<0.001
Demographic characteristics			
Age (Ref. 13 years old or younger)			
14 years old	2.53	1.08–5.95	0.033
15 years old	1.01	0.79–1.28	0.948
16 years old	1.09	0.86–1.37	0.487
17 years old	0.94	0.74–1.18	0.565
18 years old or older	0.86	0.68–1.08	0.203
Gender (Ref. Female)			
Male	1.63	1.45–1.82	<0.001
Race and Ethnicity (Ref. White)			
American Indian/Alaska Native/Native Hawaiian/Other P.I.	2.11	1.31–3.41	0.002
Asian	0.45	0.33–0.60	<0.001
Black or African American	2.63	2.27–3.04	<0.001
Hispanic/Latino	1.54	1.26–1.88	<0.001
Multiple race—Hispanic/Non-Hispanic	1.56	1.36–1.80	<0.001
Did not report	1.93	1.28–2.91	0.002
Behavioral and other factors			
Ate fruit or drank 100% fruit juices one or more times per day (Ref. Yes)			
No	1.01	0.90–1.13	0.921
Did not report	0.99	0.56–1.75	0.974
Ate vegetables one or more times per day (Ref. Yes)			
No	0.97	0.86–1.08	0.556
Did not report	0.84	0.57–1.25	0.388
Drank a can, bottle, or glass of soda or pop one or more times per day (Ref. Yes)			
No	0.95	0.82–1.10	0.476
Did not report	1.43	0.87–2.35	0.165
Drank one or more glasses per day of milk (Ref. Yes)			
No	0.98	0.84–1.13	0.740
Did not report	1.15	0.89–1.49	0.284
Ate breakfast on all 7 days (before the survey) (Ref. Yes)			
No	1.21	1.06–1.38	0.005
Did not report	1.00	0.58–1.73	0.998
Physically active at least 60 min per day on all 7 days (Ref. Yes)			
No	1.10	0.97–1.26	0.134
Did not report	0.88	0.49–1.55	0.651
Reported that their mental health was most of the time or always not good (Ref. Yes)			
No	1.09	0.96–1.23	0.173
Did not report	2.07	1.19–3.61	0.010
Got 8 or more hours of sleep (on an average school night) (Ref. Yes)			
No	0.87	0.75–1.00	0.054
Did not report	0.81	0.62–1.06	0.124
Did not sleep in their parent’s or guardian’s home (during the 30 days before the survey) (Ref. Yes)			
No	0.92	0.67–1.26	0.588
Did not report	0.54	0.28–1.02	0.058

Reference category: overweight/obese. Abbreviations: AOR—adjusted odds ratio; CI—confidence interval; Ref.—reference.

## Data Availability

Publicly available datasets were analyzed in this study. These data can be found here (2021 YRBSS National Dataset): https://www.cdc.gov/yrbs/data/index.html, accessed on 18 June 2024.

## Supplementary Materials

The following supporting information can be downloaded at: https://www.mdpi.com/article/10.3390/children12020169/s1, Table S1: Summary of variables investigated.
