# Supplementary material for: Body Weight Perception and Other Factors Associated with Overweight and Obesity in U.S. Adolescents
Source: Children (Basel). 2025 Jan 29;12(2):169. doi: 10.3390/children12020169 (PMC11854347; doi:10.3390/children12020169)
Supplement: Supplementary file 1 [file children-12-00169-s001.zip › children-3418955-supplementary.pdf]

S1 Table. Summary of variables investigated

Study variable	YRBSS identifiers	Questions asked	Response options provided	Response options analyzed
**Weight**				
BMI	Calculated Variable: QNOWT, QNOBESE OWOB	1. How tall are you without your shoes on? (in feet and inches)<br>2. How much do you weight without your shoes on? (in pounds)	1. Underweight (BMI < 18.5)<br>2. Normal weight (BMI 18.5 - 24.9)<br>3. Overweight/obese (BMI ≥ 25).	1. Underweight/normal<br>2. Overweight/obese
Self-described (perceived)	Q66	How do you describe your weight?	1. Very underweight<br>2. Slightly underweight<br>3. Normal weight<br>4. Slightly overweight<br>5. Very overweight	1. Very underweight<br>2. Slightly underweight<br>3. Normal weight<br>4. Slightly overweight<br>5. Very overweight
**Demographic factors**				
Age	Q1	How old are you?	1. 12 years old or younger<br>2. 13 years old<br>3. 14 years old<br>4. 15 years old<br>5. 16 years old<br>6. 17 years old<br>7. 18 years old or older	1. 13 years old or younger<br>2. 14 years old<br>3. 15 years old<br>4. 16 years old<br>5. 17 years old<br>6. 18 years old or older
Gender	Q2	What is your sex?	1. Male<br>2. Female	1. Male<br>2. Female
Race and Ethnicity	Calculated Variable: raceeth	What is your race?	1. American Indian or Alaska Native<br>2. Asian<br>3. Black or African American<br>4. Native Hawaiian or Other Pacific Islander<br>5. White	1. White<br>2. Asian<br>3. Black or African American<br>4. Hispanic/Latino<br>5. American Indian/Alaska Native/Native Hawaiian/Other Pacific Islander<br>6. Multiple race—Hispanic/Non-Hispanic<br>7. Did not report.
		Are you Hispanic or Latino?"	1. Yes<br>2. No	

Behavioral and other factors				
Ate fruit or drank 100% fruit juices one or more times per day	Q69	During the past 7 days, how many times did you eat fruit?	1. Did not eat/drink<br>2. 1 to 3 times<br>3. 4 to 6 times<br>4. 1 time per day<br>5. 2 times per day<br>6. 3 times per da<br>7. 4 or more times per day	1. Yes<br>2. No<br>3. Did not report
	Q68	During the past 7 days, how many times did you drink 100% fruit juices such as orange juice, apple juice, or grape juice? (Do not count punch, Kool-Aid, sports drinks, or other fruit-flavored drinks.)	1. Did not eat/drink<br>2. 1 to 3 times<br>3. 4 to 6 times<br>4. 1 time per day<br>5. 2 times per day<br>6. 3 times per da<br>7. 4 or more times per day	1. Yes<br>2. No<br>3. Did not report
Ate vegetables one or more times per day	Q73	During the past 7 days, how many times did you eat other vegetables? (Do not count green salad, potatoes, or carrots.	1. Did not eat/drink<br>2. 1 to 3 times<br>3. 4 to 6 times<br>4. 1 time per day<br>5. 2 times per day<br>6. 3 times per da<br>7. 4 or more times per day	1. Yes<br>2. No<br>3. Did not report
Drank a can, bottle, or glass of soda or pop one or more times per day	Q74	During the past 7 days, how many times did you drink a can, bottle, or glass of soda or pop, such as Coke, Pepsi, or Sprite? (Do not count diet soda or diet pop.)	1. Did not eat/drink<br>2. 1 to 3 times<br>3. 4 to 6 times<br>4. 1 time per day<br>5. 2 times per day<br>6. 3 times per da<br>7. 4 or more times per day	1. Yes<br>2. No<br>3. Did not report
Drank one or more glasses per day of milk	Q75	During the past 7 days, how many glasses of milk did you drink? (Count the milk you	1. Did not eat/drink<br>2. 1 to 3 times<br>3. 4 to 6 times<br>4. 1 time per day<br>5. 2 times per day<br>6. 3 times per da	1. Yes<br>2. No<br>3. Did not report

		drank in a glass or cup, from a carton, or with cereal. Count the half-pint of milk served at school as equal to one glass.)".	7. 4 or more times per day	
Ate breakfast on all 7 days (before the survey)	Q76	During the past 7 days, how many days did you eat breakfast?"	1. 0 days<br>2. 1 day<br>3. 2 days<br>4. 3 days<br>5. 4 days<br>6. 5 days<br>7. 6 days<br>8. 7 days	1. Yes<br>2. No<br>3. Did not report
Physically active at least 60 minutes per day on all 7 days	Q77	During the past 7 days, on how many days were you physically active for a total of at least 60 minutes per day? (Add up all the time you spent in any kind of physical activity that increased your heart rate and made you breathe hard some of the time.)	1. 0 days<br>2. 1 day<br>3. 2 days<br>4. 3 days<br>5. 4 days<br>6. 5 days<br>7. 6 days<br>8. 7 days	1. Yes<br>2. No<br>3. Did not report
Reported that their mental health was most of the time or always not good	Q85	During the past 30 days, how often was your mental health not good? (Poor mental health includes stress, anxiety, and depression.)	1. Never<br>2. Rarely<br>3. Sometimes<br>4. Most of the time<br>5. Always	1. Yes<br>2. No<br>3. Did not report
Got 8 or more hours of sleep (on an	Q86	On an average school night, how many	1. 4 hours or less<br>2. 5 hours<br>3. 6 hours	1. Yes<br>2. No<br>3. Did not report

average school night)		hours of sleep do you get?	4. 7 hours<br>5. 8 hours<br>6. 9 hours<br>7. 10 or more hours	
Did not sleep in their parent's or guardian's home (during the 30 days before the survey	Q87	During the past 30 days, where did you usually sleep?	1. In my parent's or guardian's home<br>2. In the home of a friend, family member, or other person because I had to leave my home or my parent or guardian cannot afford housing<br>3. In a shelter or emergency housing<br>4. In a motel or hotel<br>5. In a car, park, campground, or other public place<br>6. I do not have a usual place to sleep<br>7. Somewhere else	1. Yes<br>2. No<br>3. Did not report
